# Potential Prognostic Role of *SPARC* Methylation in Non-Small-Cell Lung Cancer

**DOI:** 10.3390/cells9061523

**Published:** 2020-06-22

**Authors:** Federico Pio Fabrizio, Angelo Sparaneo, Andrea Fontana, Tommaso Mazza, Paolo Graziano, Angela Pantalone, Paola Parente, Flavia Centra, Natalizia Orlando, Domenico Trombetta, Annamaria la Torre, Gian Maria Ferretti, Marco Taurchini, Concetta Martina Di Micco, Evaristo Maiello, Vito Michele Fazio, Antonio Rossi, Lucia Anna Muscarella

**Affiliations:** 1 Laboratory of Oncology, Fondazione IRCCS Casa Sollievo della Sofferenza, San Giovanni Rotondo, 71013 Foggia, Italy; fp.fabrizio@operapadrepio.it (F.P.F.); a.sparaneo@operapadrepio.it (A.S.); f.centra@operapadrepio.it (F.C.); d.trombetta@operapadrepio.it (D.T.); vm.fazio@operapadrepio.it (V.M.F.); 2 Unit of Biostatistics, Fondazione IRCCS Casa Sollievo della Sofferenza, San Giovanni Rotondo, 71013 Foggia, Italy; a.fontana@operapadrepio.it; 3 Unit of Bioinformatics, Fondazione IRCCS Casa Sollievo della Sofferenza, San Giovanni Rotondo, 71013 Foggia, Italy; t.mazza@operapadrepio.it; 4 Unit of Pathology, Fondazione IRCCS Casa Sollievo della Sofferenza, San Giovanni Rotondo, 71013 Foggia, Italy; p.graziano@operapadrepio.it (P.G.); p.parente@operapadrepio.it (P.P.); natalizia.o@alice.it (N.O.); 5 Department of Medicine, R.U. in Molecular Medicine and Biotechnology, University Campus Bio-Medico of Rome, 00128 Rome, Italy; angela.pantalone@libero.it; 6 Advanced Therapies Production Unit, Fondazione IRCCS Casa Sollievo della Sofferenza, San Giovanni Rotondo, 71013 Foggia, Italy; a.latorre@operapadrepio.it; 7 Thoracic-Surgery Unit, Fondazione IRCCS Casa Sollievo della Sofferenza, San Giovanni Rotondo, 71013 Foggia, Italy; m.taurchini@operapadrepio.it (M.T.); gm.ferretti@operapadrepio.it (G.M.F.); 8 Medical Oncology Unit, Fondazione IRCCS Casa Sollievo della Sofferenza, San Giovanni Rotondo, 71013 Foggia, Italy; e.maiello@operapadrepio.it (E.M.); doctor.dimicco@gmail.com (C.M.D.M.); arossi_it@yahoo.it (A.R.)

**Keywords:** *SPARC*, methylation, NSCLC, early-stage, squamous cell carcinoma

## Abstract

The silencing of *SPARC* (secreted protein acid and rich in cysteine) gene through methylation of its promoter region represents a common event in many solid tumors and it is frequently associated with tumor progression and an aggressive clinical outcome. Anyhow, the data concerning the epigenetic mechanism of *SPARC* deregulation and its prognostic value in lung cancer are still incomplete. We explored the aberrant methylation of *SPARC* and its effects in 4 non-small cell lung cancer (NSCLC) cell lines and 59 NSCLC tissues and correlated the methylation levels with clinical-pathological features and disease outcome of patients. In 3 out of 4 tumor cell lines high *SPARC* methylation levels were observed. An inverse correlation between the epigenetic silencing and SPARC expression was confirmed by 5-Aza-2′-deoxycytidine ((5-Aza-CdR) treatment that also significantly induced a reduction in cell viability, proliferation and tumor cell migration. In tissues, the DNA methylation levels of the *SPARC* gene were significantly lower in paired non-neoplastic lungs (NLs) and normal lungs distant from tumor (NLDTs) than in NSCLCs (*p* = 0.002 and *p* = 0.0034 respectively). A promoter hypermethylation was detected in 68% of squamous cell carcinoma (SqCCs, 17/25) and 56% of adenocarcinoma (ADCs, 19/34), with SqCC showing the highest levels of methylation. Higher *SPARC* methylation levels were significantly associated with higher mortality risk both in all NSCLCs early stage patients (Hazard Ratio, HR = 1.97; 95% Confidence Interval, CI: 1.32–2.93; *p* = 0.001) and in those with SqCC (HR = 2.96; 95% CI: 1.43–6.12; *p* = 0.003). Promoter methylation of *SPARC* gene should represent an interesting prognostic biomarker in NSCLC, with potential application in the squamous early-stage context. Further research in this setting on larger independent cohorts of lung patients with different histologies and stages of disease are warranted.

## 1. Introduction

Non-small-cell lung cancer (NSCLC) is the most common malignant epithelial tumor of the lung and accounts for approximately 85% of all new lung cancer diagnosis [1], with a 5-year overall survival (OS) rate around 18% [2]. NSCLC patients radically resected have a significant risk to progress for distant metastases of 40% overcoming the multi-step process of local stroma invasion, vasculature tumor cells dissemination and colonization at distant organs [3]. The SPARC (secreted protein acid and rich in cysteine) protein plays a central role in cancer metastasis through controlling extracellular matrix (ECM) synthesis and turnover, cell-matrix interaction and remodeling, changing of cell shape, proliferation, migration and angiogenesis [4,5]. SPARC, also known as osteonectin or basement membrane 40 (BM-40), belongs to a matricellular group of calcium-binding glycoproteins of 303 amino acids in length with a molecular mass of 43 kDa [6]. This protein contains three structural and functional domains: the acidic *N*-terminal (NT) region that binds hydroxyapatite and calcium ions, the cysteine-rich Follistatin-like domain (FS), containing Kazal-like sequences and the high-affinity with Ca^2+^-binding residues is represented by an extra-cellular domain (EC) thanks to calcium-binding motifs with “EF-hands”(EFs), [6,7].

SPARC is normally produced by capillary endothelial cells, fibroblasts, macrophages and platelets and its expression was assessed on the cell surface and within the intracellular compartment [6]. In tumors, SPARC appears to enhance growth and progression by promoting matrix remodeling and vascular network enhancement; however, it is differentially expressed in many types of cancer since its ability to inhibit and promote tumor progression depends on the cellular type, tumor staging and the complex interaction surrounding the tumor cell microenvironment [4,8]. In lung cancer, the high heterogeneity of expression in stroma and tumor cells reflects the controversial clinical value of SPARC and gives different results in cohorts of patients with different disease stages and treatments [9,10] By contrast, SPARC has a high binding affinity to albumin and its stromal expression of this protein in lung carcinoma might be considered a potential predictive biomarker in drawing albumin-bound paclitaxel to tumor cells and enhancing the ability of tumor destruction [11,12].

The expression of SPARC within NSCLC tumors appears to be influenced by epigenetic factors. The *SPARC* gene spans 26.070 kb of genomic DNA and is located on chromosome 5q33.1:151, 661,095–151,687,054 (GRCh38/hg19, December 2013), [13]. A 300 bp cytosine followed by a guanosine (CpG) rich island, ranging from exon 1 to intron 1, was firstly predicted by Sato and colleagues to be a major site of transcript *SPARC* regulation by methylation process in pancreatic cells [14]. This finding was commonly described in many solid tumors as an epigenetic event frequently associated with tumor progression and aggressive clinical outcome of patients [14,15,16,17,18,19,20,21,22].

At present, the data concerning methylation as possible epigenetic mechanisms of SPARC deregulation in lung cancer are incomplete and correlation analysis with disease clinical course in a specific subset of patients or specific therapeutic strategies is lacking. Here we hypothesized that the silencing of *SPARC* gene by aberrant methylation of its promoter CpG island during lung carcinogenesis was responsible for the downregulation of its expression in NSCLC cells. To address this hypothesis, we evaluated *SPARC* mRNA and promoter methylation levels in a collection of NSCLC cell lines and primary lung NSCLCs from surgically resected patients. Finally, we assessed the association between molecular and clinical-pathological findings and disease outcomes.

## 2. Materials and Methods

### 2.1. Cell Lines

The human NSCLC cell lines, A549, H2228, H1573, H460 and the non-malignant lung epithelial and lung fibroblast normal cell lines, NL20 and MRC5, were grown in RPMI-1640 medium (Euroclone Spa, Pero, Milan, Italy) supplemented with 10% fetal bovine serum (FBS) and 1% Penicillin/streptomycin and incubated in 5% CO_2_ at 37 °C.

### 2.2. Patients and Tumor Tissue Samples

A total of 59 NSCLC Formalin-fixed paraffin-embedded (FFPE) samples (25 squamous cell carcinoma (SqCC) and 34 adenocarcinoma (ADC) from NSCLC patients (with 19/59 paired non-neoplastic lung (NL) tissues available) and 11 unpaired non-neoplastic lung tissues (NLDT Normal Lung Distant from Tumor) were obtained at Fondazione IRCCS “Casa Sollievo della Sofferenza” from years 2004 to 2014. The latest information on vital status and disease progression was obtained in 2018. At follow-up, the vital status of study patients was ascertained either by telephone interview with the patient or his/her relatives or by queries to the registry office of cities of residence. The patients’ clinical and pathological features including Tumor-Node-Metastasis (TNM) staging system, lymph nodes diffusion, grading, age, gender and follow-up data were collected at the date of hospitalization.

An additional, independent learning cohort of 21 paired NL/NSCLC used for methylation analysis on paired non-neoplastic/NSCLC and immunohistochemistry investigations was also obtained at Fondazione IRCCS “Casa Sollievo della Sofferenza” from 2017 to 2018, without the collection of follow-up information.

The study was conducted in accordance with the Declaration of Helsinki and the protocol was approved by the Ethics Committee of Fondazione IRCCS Casa Sollievo della Sofferenza (Prot 76/CE). 

### 2.3. DNA and RNA Extraction

Genomic DNA was extracted from each cell line and FFPE samples by using the standard Phenol-Chloroform procedure and the GeneRead DNA FFPE Kit (Qiagen, Hilden, Germany), respectively. Total RNA was extracted from cultured cells with Trizol reagent (ThermoFisher Scientific, Waltham, MA, USA) following the manufacturer’s instructions. DNA and RNA concentrations were measured by NanoDrop spectrophotometer ND-1000 and fluorimeter Qubit (ThermoFisher Scientific, Waltham, MA, USA).

### 2.4. Cell Culture and 5-Aza-2′-Deoxycytidine (5-Aza-CdR) Treatment

The A549 cell line was seeded in six-well culture dishes and incubated in fresh culture medium with 5 μM of the demethylating agent 5-Aza-2′-deoxycytidine (Sigma-Aldrich, St. Louis, Missouri, USA) for 24 and 48 h. Cells were then harvested for genomic DNA and total RNA extraction to demonstrate if the treatment with the demethylating agent was able to restore *SPARC* mRNA expression levels in this cell line. 

### 2.5. Proliferation, Viability, Migration and Invasion Assays

To evaluate cell proliferation, A549 cells were cultured in a six-well plate for 24 h. Subsequently, cells were treated with 5 µM of 5-Aza-2′-deoxycytine for 24 h and 48 h. The number of cells adherent onto each well was determined using an automatic cells counter after being treated with 0.25% trypsin-EDTA (Ethylenediaminetetraacetic Acid) solution and trypan blue staining. The number of replicates well was 4 (*n* = 4).

Cell migration was evaluated by a scratch wound assay. Confluent monolayer of A549 seeding on six-wells was scratch wounded with a 200 µL micropipette tip and treated with 5 µM of 5-Aza-2′-deoxycytidine. Debris and dislodged cells were removed by washing cells with PBS. Fields of wound closure were taken immediately after scratching and at 24 and 48 h post- wounding using inverter Microscope (Nikon, Minato, Tokyo, Japan). Image J software was used to measure scratch gap, calculating the ratio of the scratch gap at the given point in time and the original gap, 0 h. Al least four-microscope fields were counted for each condition.

Prestoblue assay (Thermo Scientific, USA) was carried out to assess cell viability after 5-Aza-2′deoxycytidine (Sigma-Aldrich) treatment. A549 cells were seeded in a 96 well plate at a concentration of 8 × 10^3^ cells per well. On the next day, 5 µM of 5-Aza -2′ deoxycytidine was added to wells for 24 and 48 h. Cell viability was measured after 24 h and 48 h of treatment adding prestoblu solution for 3 h. Synergy HT multimode microplate reader (BioTek Instrument, Winooski, VT, USA) was used to fluorescence acquisition following the manufacturer’s instructions.

The invasion assay was performed by using transwells with 8μm porous membrane coated with an invasion matrix containing Type IV Collagen, Human Laminin, and Gelatin diluted in PBS. 8 × 10^5^ A549 cells were seeded in 24 multi-wells onto transwell and treated with 5 µM of 5-Aza -2′-deoxycytidine for 24 h and 48 h. Each experiment was performed in triplicate. The invasion assay was stopped after 24 h and 48 h and cells were fixed in formalin 10% for 10 min before staining using crystal violet for 15 min. For each well, ten random fields were counted, and the average number of cells was determined [23].

### 2.6. Immunoistochemistry (IHC)

From the FFPE of the learning cohort of 21 NSCLCs, 3 µm sections were selected for IHC analysis and incubated with 1:200 rabbit monoclonal anti-SPARC antibody (D10F10, Cell Signaling, Danvers, MA) for 60 min at RT. The primary antibody was detected by using a commercially available detection kit (EnVisionTMFLEX+, Dako, Glostrup, Denmark) following the manufacturer’s protocol and diaminobenzidine as chromogen.

Slides were washed with Tris-buffered saline (TBS, 0.1 M, pH = 7.4), 3–5 times after each step. Finally, the sections were counterstained with Mayer’s hematoxylin and mounted with Biomount (BIO-OPTICA, Milan, Italy). In the negative control tissue sections, the primary antibody was replaced by isotype specific non-immune rabbit IgG and small peritumoral vessels were used as internal positive control for SPARC expression. The immunoreactivity was assessed in the whole neoplastic area of the tissue section. SPARC protein expression was scored as positive if membrane/cytoplasm reactivity was observed in tumor cells.

### 2.7. Reverse Transcription-Polymerase Chain Reaction (RT-PCR)

The RT-PCR was used to monitoring the *SPARC* transcript level variations during the 5-Aza-2′-deoxycytidine treatment. First-strand cDNA synthesis from 500 ng of total RNA extracted from cell line was carried out with SuperScript III First-Strand Synthesis (Thermo Fisher, Invitrogen Division, Carlsbad, CA, USA) using TaqMan™ Gene Expression Assay mixture containing 2.5× TaqMan^®^ Universal PCR Master Mix (Thermo Fisher, Life Technologies division), 250 nM of TaqMan probe and 1 μL of template cDNA or plasmid product (serial dilutions). The Primer/Probe sets for *SPARC* and *RPLPO* genes expression were as follows: Hs00234160_m1 and 4326314E (Thermo Fisher, Life Technologies). The real-time quantitative RT-PCR was run on ABIPRISM 7900HT Sequence Detection System (Thermo Fisher, Life Technologies Division). For the quantification of gene expression, the SPARC values were normalized to the expression of the housekeeping *RPLPO* gene as the ratio marker. Expression transcript levels were calculated by the relative quantification method using plasmid dilutions between 10^6^ and 10^2^ copies of pSC-A plasmid standard curves (Stratagene, Milan, Italy) and resulted in plasmid copy number.

### 2.8. Sodium Bisulfite Conversion and Quantitative Methylation Specific PCR Analysis (QMSP)

Methylation levels of the CpGs mapped in the *SPARC* promoter region were determined in cell lines and tissue samples by using QMSP starting from 1 μg of genomic DNA treated with sodium bisulfite using Epitect Bisulfite kit (Qiagen, MD, USA). Primer sequences of *SPARC* promoter region were 5′-ATATTTTCGCGGTTTTTTAGA-3′ (forward) and 5′-AACGACGTAAACGAAAATATCG-3′ (reverse), whereas the unmethylated promoter region of the ACTB as reference gene was amplified using the 5′-GGTGATCGAGGAGGTTTAGTAAGT-3′ forward and 5′-AACCAATAAAACCTACTCCTCCCTTAA-3′ reverse primers. Probe sets used for *SPARC* and *ACTB* were as follows: FAM-AGCGCGTTTTGTTTGTCGTTTGTTTG-TAMRA and FAM-ACCACCACCCAACACACAATAACAAACACA-TAMRA, respectively. Calibration curves for both target and reference genes were obtained by using serial dilutions (90–0.009 ng) of commercially available fully methylated DNA (CpGenome Universal Methylated DNA, Millipore). Amplification reactions were performed in triplicate in 384-well plates and in a final volume of 10 μL that contained 50 ng of bisulfite-modified DNA, 100 pmol/L concentrations of forward and reverse primers, 200 nM probe and ROX (6-carboxy-X-rhodamine) Reference Dye, 0.6 U of platinum Taq polymerase (Invitrogen, Frederick, MD, USA), 25 mM concentrations of dNTPs (deoxynucleoside Triphosphates) set. Reaction conditions were used by the following profile: 95 °C for 3 min, followed by 50 cycles at 95 °C for 15 s and 60 °C for 1 min and were carried out on ABIPRISM 7900 Sequence detection system (Applied Biosystems, Foster City, CA, USA) and were elaborated by software development specification (SDS) 2.1.1 version(Applied Biosystems). *SPARC* methylation levels were calculated as the ratio between the average value of triplicates of *SPARC* and the average value of triplicates of *ACTB* for each sample (Supplemental Figure S1).

### 2.9. Mutation Screening of Epidermal Growth Factor Receptor Tyrosine Kinase (EGFR) and Kirsten Rat Sarcoma Viral Oncogene Homolog (KRAS) Genes by Sanger Sequencing

DNA from tissues was PCR-amplified. Different coding hot spot regions were analyzed for *EGFR* (exons 18–21) and *KRAS* (exon 2) genes. PCR products were analyzed for the presence of mutations by using direct sequencing on ABIPRISM 7900HT Sequence Detection System (Thermo Fisher, Life Technologies division) 3100 (Life Technologies) and Sequencing Analysis Software v.3.7.

### 2.10. TCGA Data Analysis

Methylation and expression data of Lung Squamous Cell Carcinoma (TCGA-LUSC) and Lung Adenocarcinoma (TCGA-LUAD) datasets were directly pulled down from University of California Santa Cruz (UCSC) Xena public data hubs.These data include *n* = 877 (LUAD) and *n* = 765 (LUSC) affected patients. In particular, DNA methylation data were generated by Infinium Human Methylation 450K BeadChip microarrays and are stored in the Pan-Cancer Atlas Hub; gene expression data were obtained by RNA-Seq experiments and are available from the UCSC Toil RNAseq Recompute Compendium. Methylation data were available as beta-values, while expression data were available as TPM-normalized reads counts.

### 2.11. Statistical Analysis

Patients’ clinical and histological characteristics were reported as mean ± standard deviation (SD) or absolute frequencies and percentages for continuous and categorical variables, respectively. The discriminatory power of the *SPARC* QMSP assay was assessed by estimating the Area under the Receiver Operating Characteristics (ROC) curve (AUC). The optimal cut-off of the *SPARC* methylation levels, which best discriminated all NSCLC from NLDT tissues, was determined as the one which jointly maximizes sensitivity and specificity measures in the ROC space (i.e., achieving the highest Youden’s index). Such cut-off was used to determine the presence of methylation in the tissue samples. Boxplots of *SPARC* promoter methylation levels among three different tissue types (i.e., ADC, SqCC and NLDT) were also reported. Patients’ characteristics were compared between the two methylated groups using Mann-Whitney U test or Fisher exact test for continuous and categorical variables, respectively. Moreover, the association between *SPARC* methylation levels and categorical patients’ characteristics was assessed by Mann-Whitney U test (or Kruskal-Wallis as appropriate) whereas the correlation with continuous characteristics was assessed by Spearman correlation coefficient. The individual overall follow-up time was defined as the time between the date of tumor diagnosis and the occurrence of the death for any cause (OS) whereas the individual time to disease progression was defined as the time between the date of tumor diagnosis and the occurrence of the first disease progression (progression-free survival, PFS). For patients who did not experience any event, their individual follow-up time was defined as the date between tumor diagnosis and the end of observational period (last available date). Yearly incidence rate was defined as the number of events divided by the number of person-years × 100. To assess the association between *SPARC* methylation levels (and status) and disease outcomes (i.e., OS, PFS), time-to-event analysis was performed by univariable Cox proportional hazards regression models and risks were reported as hazard ratios (HR) along with their 95% confidence interval (CI). This analysis was performed for all NSCLC patients and within early stage tumor NSCLC patients only, according to their tumor histology. When *SPARC* methylation levels were considered as the main covariate into the Cox model, HRs were reported for each unitary increase in one SD of such methylation levels. The assumption of proportionality of the hazards and the assumption of risks linearity at each SD increase of the *SPARC* methylation levels was assessed by Kolmogorov-type supremum test [24]. Kaplan-Meier curves were also shown with respect to *SPARC* methylation status for OS and PFS outcomes at issue. Moreover, to provide an efficient non-parametric analysis of time to event data, Random Survival Forest (RSF) was performed [25]. RSF is an extension of Breiman’s Random Forest [26] techniques to survival settings: it is a robust, non-linear technique (it does not require any distributional assumptions on covariate relation to the response) that optimizes predictive accuracy by fitting an ensemble of trees to stabilize model estimates. Variable dependence plots, which show the relationship between the RSF predicted out-of-bag outcome responses (i.e., OS and PFS) and *SPARC* methylation levels, were produced.

Methylation level comparison between tumors and paired non-neoplastic tissues was made by using the Wilcoxon Signed rank test.

Correlation between *SPARC* mRNA expression and all individual beta-values of *SPARC* in the TCGA datasets was assessed using Pearson’s correlation coefficient. Similarly, an overall assessment of correlation was calculated aggregating the beta-values of all CpGs (average).

All statistical analyses aimed to search for correlation between *SPARC* methylation, and clinical-pathological features and disease outcomes were performed using SAS Release 9.4 (SAS Institute, Cary, NC, USA). Plots were performed using R Foundation for Statistical Computing (version 3.6, packages: randomForestSRC, ggRandomForests, ggplot2, gridExtra).

For in vitro experiments, the relationship between methylation and *SPARC* expression, differences in viability, proliferation and migration of cells were examined using Student’s t-test and analyzed with GraphPad Prism 5 (GraphPad Software, Inc., La Jolla, CA, USA). 

All results were deemed statistically significant when *p* is < 0.05.

## 3. Results

### 3.1. SPARC CpG Island Prediction and QMSP Assay Optimization

**Figure 1:**
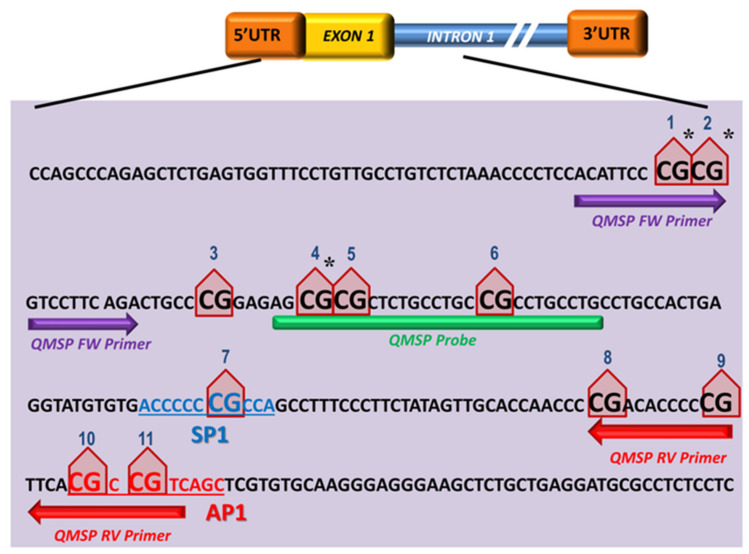
Sequence of SPARC promoter region (−29 bp to +191 bp) located spanning from exon 1 to intron 1 of the gene. Eleven CpGs are marked as red triangle shape and numbers are given from 5′ to 3′ of positive strand of the *SPARC* gene. The QMSP amplified region counts 111 bp (Forward in purple arrow, Probe in green segment and Reverse in red arrow). The letters colored and underlined indicate SP1 (blue) and AP1 (red) binding consensus sequences, respectively. * indicates the CpGs included in the 450 K array.

The SPARC methylation status was assessed by designing a primers/probe set that amplifies the CpG region in the gene promoter region showing the highest frequency of methylation and that contains a consensus sequence for the transcriptional Sp1 and AP1 regulatory elements [18]. The entire DNA sequence, including the upstream region of transcription start sites and the CpG promoter island of SPARC, were retrieved using the UCSC database and the Methprimer software (http://www.urogene.org/cgi-bin/methprimer2/MethPrimer.cgi) was used to map the CpG islands of the SPARC promoter and design the QMSP assay. The putative hypermethylated CpG-rich site was restricted close to the SPARC promoter region (from −29 bp to +191 bp) around the transcriptional start site (TSS) and included 11 CpGs [22] (Figure 1).

### 3.2. Aberrant SPARC Methylation Is a Frequent Event in Primary NSCLCs

The *SPARC* methylation levels were firstly evaluated on DNA obtained from a learning cohort of 21 paired lung non-neoplastic/NSCLC tissues (Supplementary Table S1) and a statistically significant difference in methylation levels was detected between paired non-neoplastic and tumor tissues (*p* = 0.006; Wilcoxon signed rank test).

The epigenetic silencing of *SPARC* by methylation was then evaluated on DNA bisulfite-treated obtained from 59 surgically resected NSCLCs (19/59 non-neoplastic/tumor paired) and 11 normal lung tissues from non-neoplastic patients (NLDTs). No difference in the *SPARC* methylation levels was observed between NLDT and NL of paired available 19 NSCLC tissues, whereas ordered differences were observed in the *SPARC* methylation level from the NL and the NLDT samples to the NSCLC samples (*p* = 0.002 and *p* = 0.0034 respectively; Wilcoxon signed rank test). The same significant difference between paired NL/NSCLC was observed if considering SqCC and ADC histology alone (*p* = 0.018 and *p* = 0.001 respectively; Wilcoxon signed rank test).

**Figure 2:**
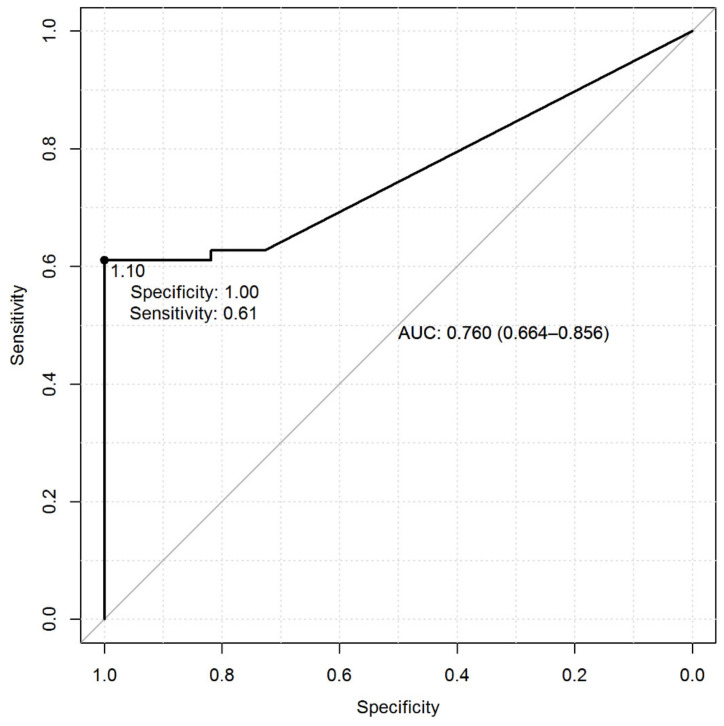
Receiver Operating Characteristic (ROC) curve calculated from *SPARC*/*ACTB* (Actin β) promoter methylation ratio in normal lung distant from tumor (NLDT) paired with NSCLCs and assessed by QMSP. The ROC curve shows the sensitivity (y axis) and specificity (x axis), based on the comparison between tumor (ADC and SqCC) and NLDT (Normal Lung Distant from Tumor) groups (Mann-Whitney *p*-value, *p* < 0.001).

Specifically, the methylation levels achieved a discriminatory power of 0.76 (AUC) to distinguish NSCLCs from NLDTs and the value of 1.10 resulted in the optimal threshold (Figure 2). When levels were categorized with respect to this cut-off, they achieved a sensitivity of 61% and a specificity of 100%. The presence of methylation was declared when methylation levels were greater than or equal to the optimal cut-off.

**Figure 3:**
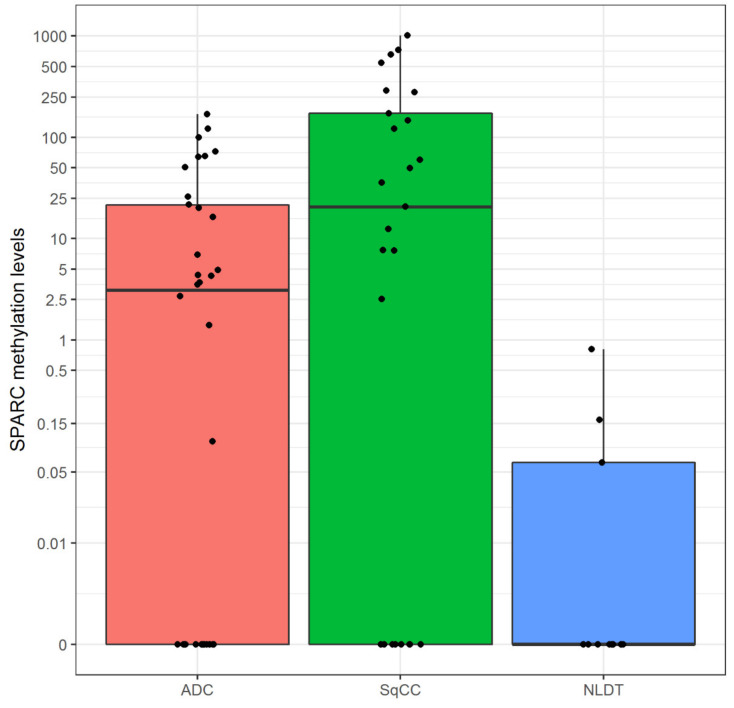
Boxplots of global *SPARC* promoter methylation among the three phonotypical groups (pink for ADC, green for SqCC and blue for NLDT). The following five number summaries were reported into each box plot: minimum, first quartile, median, third quartile, and maximum. The central rectangle spans the first quartile to the third quartile (i.e., the interquartile range or IQR). The segment inside the rectangle shows the median and “whiskers” (above and below each box) show the locations of the minimum and maximum.

*SPARC* methylation was detected in 36 (58%) of resected NSCLCs. Similar methylation frequency was found between ADC (19/34, 56%) and SqCC (17/25, 68%) although slightly higher in the SqCC type (Figure 3). In the SqCC subgroup, the median methylation level was 20.6 plasmid copy numbers in a range between 0.00 and 1016. Instead, in the ADC subgroup, the median methylation level was 3.1 plasmid copy numbers in a range between 0.00 and 170. 

### 3.3. Hypermethylation of SPARC Gene in NSCLC Cell Lines and Association with Reduced SPARC mRNA Level

**Figure 4:**
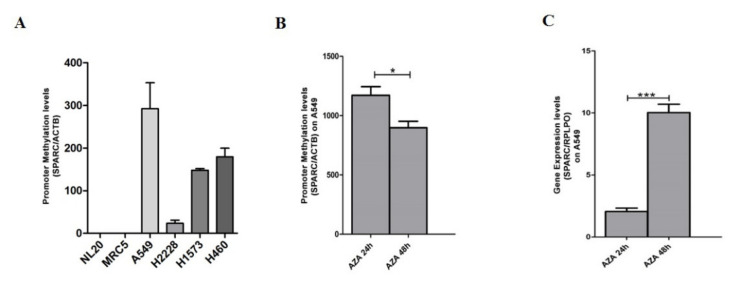
(**A**) Representative QMSP analysis showing the comparison between the promoter methylation levels of *SPARC* in NSCLC cell lines (A549, H2228, H1573, H460). Two normal lung cell lines NL20 and MRC-5 were used as negative controls. Changes in (**B**) *SPARC* promoter methylation levels detected by QMSP and (**C**) *SPARC* mRNA transcript levels by quantitative real-time (RT-qPCR) in the A549 cell line before and after treatment with 5µm of 5-azacytidine (5-aza-Dc, AZA) at 24 (AZA 24 h) and 48 h (AZA 48 h). Error bars indicate the standard deviation of three different experiments. * *p* < 0.05, *** *p* < 0.001 (*t*-test).

The methylation status of the *SPARC* gene evaluated by QMSP firstly in four NSCLC cell lines: A549, H2228, H1573 (ADC) and H460 (large cell carcinoma, LCC) and in the two non-neoplastic cell lines NL20 and MRC-5. Variable levels of methylation of *SPARC* were observed in the tumor cell lines ranged as follows: 0–292.5 ± 60.6 (A549), 23.4 ± 7.4 (H2228), 148 ± 6.8 (H1573) and 179.3 ± 20.4 (H460), (Figure 4A), whereas in normal cells no methylation was detected. The downstream effect of epigenetic silencing was therefore investigated in hypermethylated A549 cell lines under 5-Aza-2′-deoxycytidine treatment (5 µM) to demonstrate if the demethylating agent was able to restore SPARC mRNA level. A progressive rescue at *SPARC* transcript levels after 24 and 48 h (*p* < 0.001, *t*-test) and a decreased *SPARC* promoter methylation after 24 and 48 h (*p* < 0.05, *t*-test) was observed (*p* = 0.02, Pearson correlation) (Figure 4B,C).

**Figure 5:**
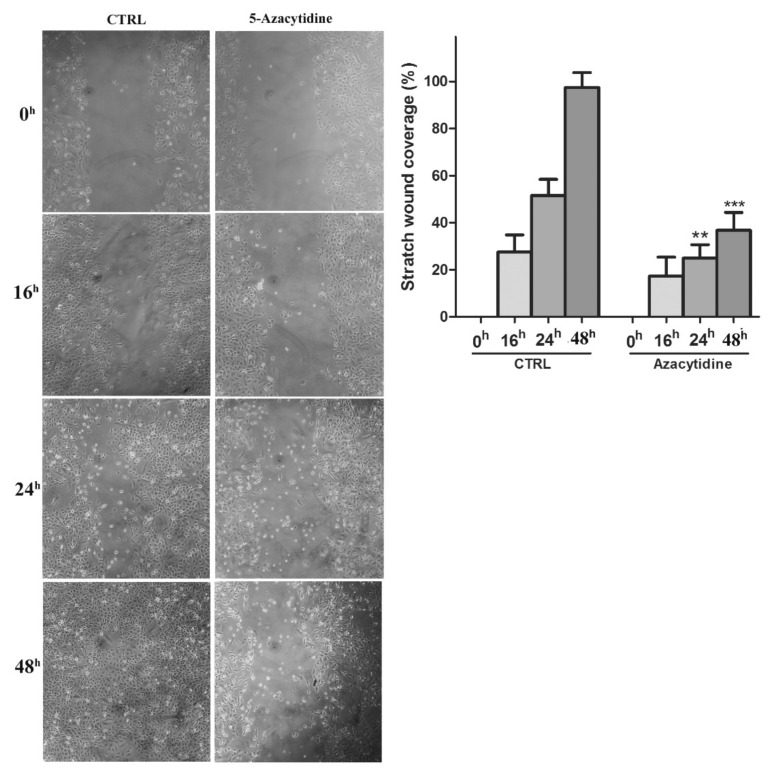
Cell migration (scratch wound healing assay). Representative images are shown from four independent experiments. Histograms show coverage percentage after 5-Aza-Cdr treatment for both control and treated samples. Values of percentage wound coverage ± SEM (*n* = 4). ** *p* < 0.01; *** *p* < 0.001 (*t*-test).

**Figure 6:**
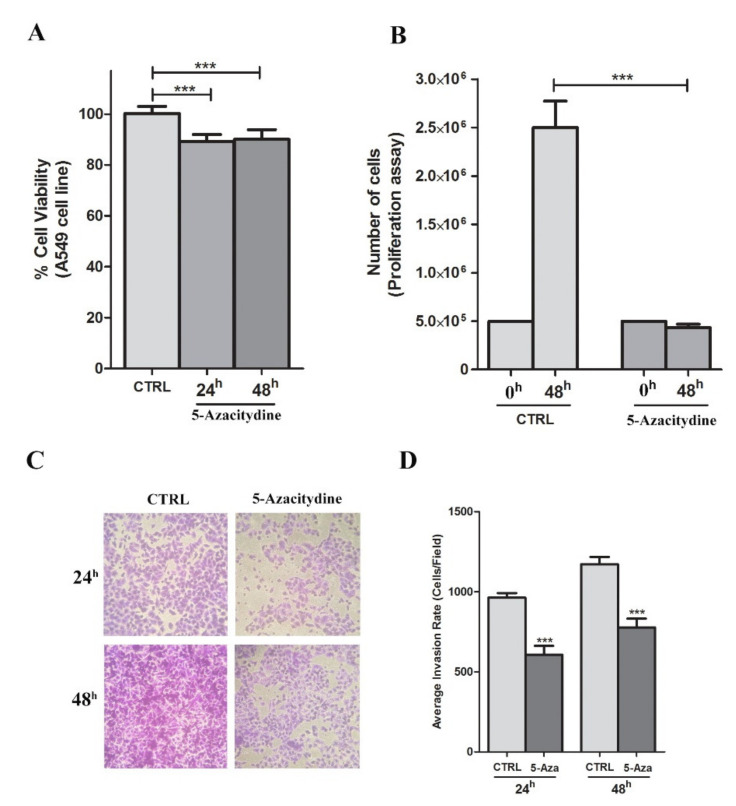
The effects of 5-Aza-Cdr treatment on viability, proliferation and migration of A549 cell line. (**A**) Results from the viability assay performed using PrestoBlue assay after 5-Aza-Cdr treatment. Data are expressed as mean ± SEM vs. ctrl. (**B**) Results from the proliferation assay after 5-Aza-Cdr treatment. Data are expressed as mean ± SEM (*n* = 4). *** *p* < 0.001 vs. untreated samples (*t*-test). (**C**) Photographs of representative fields of stained cells that migrated through the membrane during the invasion assay after 24 and 48 h of incubation. (**D**) Columns represent the average of the number of cells per field of at least ten fields in three independent experiments of invasion assay. Data are expressed as mean ± SEM. *** *p* < 0.001 vs. untreated samples (*t*-test).

Changes in proliferation, invasion and migration after incubation with 5-Aza-Cdr were also examined. In A549 cell line that resulted methylated for *SPARC* gene, the cell migration significantly decreased after 5-Aza-Cdr treatment at 24 h and 48 h (*p* < 0.001, *t*-test) (Figure 5). Similarly, the cell proliferation and invasion were also decreased after 48 h of 5-Aza-Cdr treatment with about 5-fold and 1.5 fold decrease, respectively (*p* < 0.001, *t*-test) (Figure 6B–D). These results were concordant with the next set of observations, demonstrating a 10% reduction in cell viability 5 µM 5-Aza-Cdr treatment (*p* < 0.001, *t*-test) (Figure 6A).

**Figure 7:**
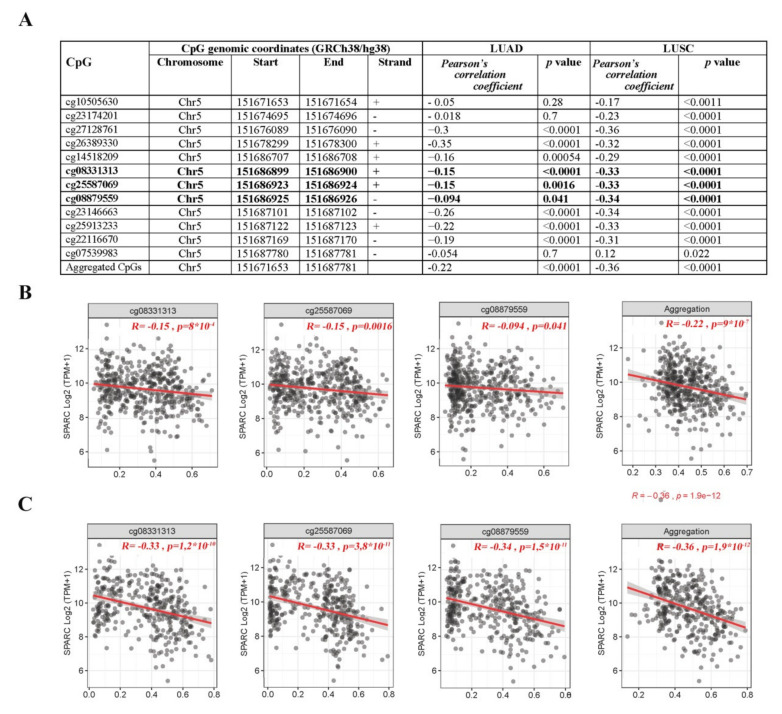
Correlation analysis between *SPARC* methylation (450 K BeadChip array, Beta-values) and expression (RNA-Seq, Transcripts per million, TPM-normalized read counts) values from TCGA-LUAD and TCGA-LUSC datasets. (**A**) Probes ID, chromosomal location, Pearson’s correlation coefficient and significance level. Bold lines refer to the three CpGs located in the *SPARC* island included in the QMSP assay. Scatter plots between *β*-values (x-axis) and expression values of SPARC in (**B**) LUAD and (**C**) LUSC TCGA datasets and concerning the CpGs located in the promoter region subjected to QMSP analysis in our study cohort.

The functional effect of *SPARC* promoter methylation on its expression was analyzed in two independent TCGA datasets of 877 lung adenocarcinomas (LUADs) and 765 lung squamous cell carcinomas (LUSCs). The *SPARC* gene has a 300 bp CpG rich island, ranging from exon 1 to intron 1, extending from the promoter region to intron 1, that is recognized by three out of twelve probes (denoted as 1 to 12) present on the Illumina Human-Methylation450 Bead Chip, all near the transcription start site of the *SPARC* gene. A highly significant inverse correlation between aberrant *SPARC* promoter methylation and its mRNA expression was found in both LUAD and LUSC (Figure 7). Specifically, in LUAD samples almost all CpG were inversely correlated with the expression of *SPARC* (except cg07539983 and cg08879559), whereas in LUSC all but cg07539983 CpGs were inversely correlated with the expression of *SPARC* (Supplemental Materials Figure S2).

**Figure 8:**
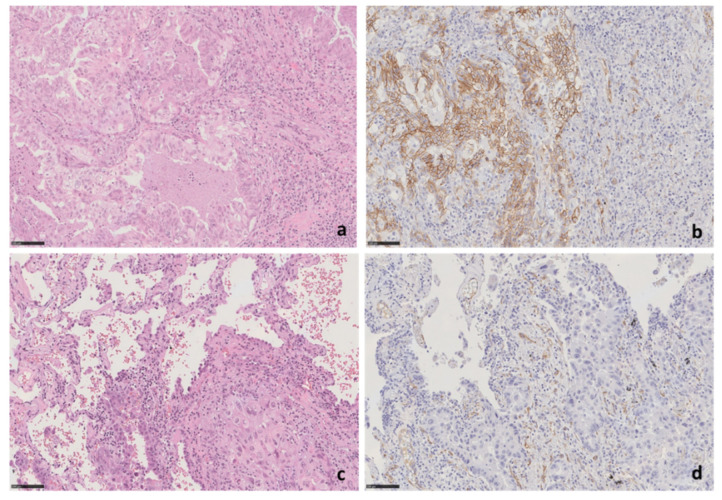
SPARC protein expression by immunohistochemical analysis. (**a**,**b**) Morphological features of case (LCCH-773) of lung adenocarcinoma, negative for *SPARC* promoter methylation, showing strong SPARC expression. (**a**) H&E staining, original magnification 20×; (**b**) SPARC staining, 20× original magnification. (**c**,**d**) Morphological features of case LCCH-889 of lung adenocarcinoma that does not show SPARC expression in tumor cells. (**c**) H&E staining, original magnification 20×; (**d**) SPARC staining, 20× original magnification. Small peritumoral vessels represent positive internal control. H&E—Hematoxylin and eosin stain. SPARC protein expression by immunohistochemical analysis. (**a**,**b**) Morphological features of case (LCCH-773) of lung adenocarcinoma, negative for *SPARC* promoter methylation, showing strong SPARC expression. (**a**) H&E staining, original magnification 20×; (**b**) SPARC staining, 20× original magnification. (**c**,**d**) Morphological features of case LCCH-889 of lung adenocarcinoma that does not show SPARC expression in tumor cells. (**c**) H&E staining, original magnification 20×; (**d**) SPARC staining, 20× original magnification. Small peritumoral vessels represent positive internal control. H&E—Hematoxylin and eosin stain.

To assess in tissues a possible correlation between the SPARC protein levels in NSCLC cells and the epigenetic silencing of the *SPARC* gene, the learning cohort of paired non-neoplastic/NSCLC tumors were also analyzed by immunohistochemistry. The SPARC immunoreactivity in tumor cells was observed only in the cytoplasm/cellular membrane of one case out of 21 NSCLCs (about 5%) having adenocarcinoma histology (Figure 8). By consequence, no significant correlation between epigenetic silencing and SPARC protein levels was possible. Nevertheless, this sample size was insufficient to investigate the presence of a possible correlation between *SPARC* methylation and protein expression by IHC in tissues.

### 3.4. SPARC Hypermethylation Is Associated with Higher Mortality Risk in SqCC Ratients

Patients’ clinical-pathological features are shown in Table 1. The mean age of the analyzed patients at the time of diagnosis was 67.7 ± 8.4 years (ranging from 44 to 85 years). The median follow-up time for NSCLC patients was 52 months (ranging from 0 to 150 months). In ADC, the median follow-up time was 49.9 months, whereas in SqCC it was 66.0 months. The estimated mortality rates were 11.0 and 10.5 events per 100 person-years for ADC and SqCC patients, respectively. The median time to disease progression in ADC was 23.1 months whereas in SqCC it was 57.9 months. The estimated disease progression rates were 19.6 and 7.7 events per 100 person-years for ADC and SqCC patients, respectively.

Table 2 and Table 3 summarizes patients’ clinical-pathological features according to *SPARC* methylation status and levels, respectively. In the latter, methylation levels were reported with respect to each feature in ADC and SqCC patients, separately. No statistically significant associations were found between methylation (status and levels) and any clinic-pathological feature both in the whole sample of NSCLC patients and within tumor histology groups.

**Figure 9:**
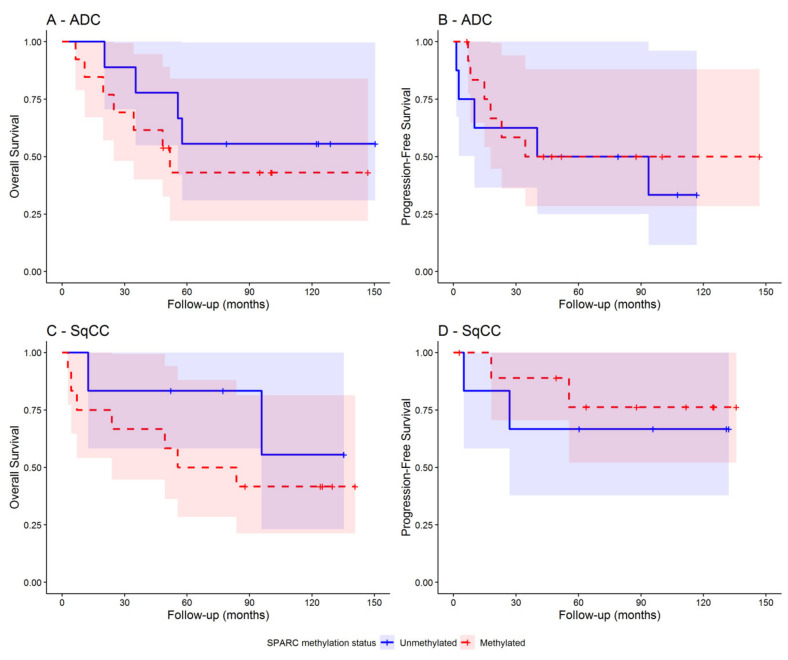
Kaplan-Meier curves showing the overall survival and disease progression-free survival in early tumor stage (I–II) NSCLC patients with ADC (panels **A** and **B**) and SqCC (panels **C** and **D**), respectively. Separate curves were reported for patients with methylated (red curve) and unmethylated (blue curve) SPARC gene promoter. The presence of *SPARC* methylation was defined for SPARC methylation levels ≥ 1.10.

**Figure 10:**
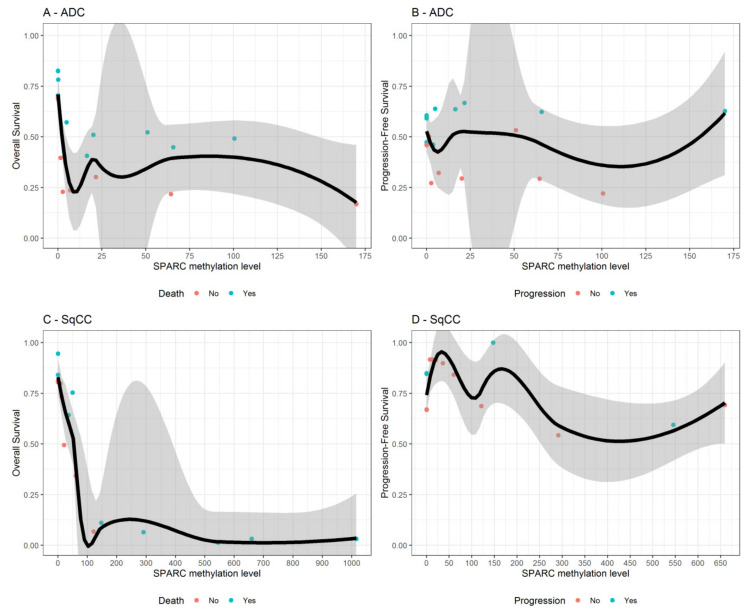
For NSCLC patients with early tumor stage only, variable dependence plots show the relationship between *SPARC* methylation levels and both the overall-survival and progression-free survival, estimated from Survival Random Forest, for patients with ADC (panels **A** and **B**) and SqCC (panels **C** and **D**), respectively. Individual cases are marked with blue (alive or censored) and red circles (events). Loess smooth curve with shaded 95% confidence band indicates decreasing survival with increasing *SPARC* methylation levels.

As shown in Table 4, higher *SPARC* methylation levels were significantly associated with a higher mortality risk both in all NSCLC patients (HR = 1.46; 95% CI: 1.07–2.00; *p* = 0.018) and in SqCC patients (HR = 2.04; 95% CI: 1.21–3.45; *p* = 0.008). The risk became much higher within NSCLC patients with early tumor stage (HR = 1.97; 95% CI: 1.32–2.93; *p* = 0.001), especially in those also with SqCC (HR = 2.96; 95% CI: 1.43–6.12; *p* = 0.003). Assumption of proportional hazards and risks linearity were met. In contrast, *SPARC* methylation status did adequately discriminate patients with lower and higher disease outcomes risk both in the overall sample and within those with early tumor stage (Figure 9). Interestingly, among NSCLC patients with early tumor stage (I–II), the estimated overall survival was dramatically reduced from 80% to 10% when *SPARC* methylation levels passed from 0 to 300 and thereafter tended toward 0% for greater values (Figure 10, panel C). A similar pattern was found when looking at all NSCLC patients Supplemental Figure S3, panel C).

To attempt a possible co-occurrence of the methylation status of *SPARC* gene and other driver molecular lesions in NSCLCs, we tested all cohort for the *EGFR* and *KRAS* genes. *KRAS* mutations were identified in five cases (20%), (Supplemental Table S2), but no significant correlation between *KRAS* mutated status and methylation of *SPARC* gene was found.

## 4. Discussion

In lung cancer, the effect of the epigenetic modulation of *SPARC* is poorly investigated; by contrast, more data are available about the role of SPARC protein in the neoplastic lung context, where it appears heterogeneously expressed in NSCLC tissues, with predominant localization in the tumor-associated-stroma. High levels of SPARC are often found to be associated with tumor malignity parameters, such as necrosis and hypoxia condition and strongly correlated with poorer post-operative OS [10,27]. High stromal expression of SPARC appears more frequent in the SqCCs than in ADCs and should be considered a predictive marker for the selection of patients likely responsive to nab-paclitaxel treatment [12]. High levels of SPARC are rarely observed within NSCLC cells, but in few scientific reports, this biological condition was associated with longer survival of patients, independent of any treatment [10,28].

*SPARC* gene is not considered a classical tumor suppressor gene since it does not exhibit point mutations or deletions that may be responsible for the variations in its expression [29]. This suggests that other different modulatory mechanisms may exist. We demonstrated here that aberrant methylation of *SPARC* promoter region should be considered a frequent event in lung cancer cell lines from different histologies and NSCLC patients. We observed that variable methylation levels were present in NSCLC cell lines and in NSCLC primary site of tumors, but absent in normal cell lines and in the normal lung tissues tested that showed low levels of methylation. Moreover, when paired tumor and normal tissues were compared, variable levels of methylation were found with no correlation with smoking habits of patients, but significantly lower in normal than in cancerous NSCLC tissues. As previously reported, it is possible that the presence of low methylation levels in some of the non-neoplastic lung epithelium may represent an early epigenetic event that predisposes patients to develop lung cancer [30]. A clear distinction between *SPARC* methylation levels in early ADC and SqCC not emerged due to the small size of the cohort, so the possible existing link of this epigenetic event to a specific NSCLC histology remains unsolved and requires feature investigations.

In tumor tissues, we found *SPARC* promoter hypermethylation in the 58% of NSCLCs analyzed; in particular, it was detected in 68% of SqCCs and 53% of ADCs. Even though the distribution of global *SPARC* methylation between the two histologies has a similar frequency in the two ADC and SqCC histologies, methylation levels appeared to be higher in tissues with squamous histology. This finding is concordant with the observation that *SPARC* protein variation levels were frequently observed in the squamous histology, where SPARC protein expression appears mainly expressed in fibroblasts and the cellular matrix, but absent (<5%) in tumor cells. Evidence about this is lacking, with only a few reports available [27,31]. When expressed within the tumor, SPARC could be protective and possibly buffer the aggressiveness of the tumor itself, highlighting the tissue-specific functions of SPARC in assisting crosstalk at the tumor-stroma interface [27,31].

A rare expression of the SPARC protein in NSCLC cells was also observed in our learning cohort (only 1/21 cases); as a consequence, it was not possible to prove a statistically significant correlation between methylation of SPARC and its expression in tumor cells of lung tissues; moreover, SPARC protein expression data are not available on the TCGA datasets. Despite this, a good inverse correlation between *SPARC* methylation and its mRNA levels under 5-Aza-CdR treatment was observed in the A549 cell line, thus corroborating the idea of a regulatory role of some CpGs at the *SPARC* promoter region among those ones that Gao and colleagues have identified and called CpG region 1 (which contains CpG sites 1–7 and includes SP1 binding consensus sequence) and CpG region 2 groups (CpG sites 8–12, which includes AP2 binding consensus sequences) [18,32]. 

In support of our in vitro studies, the same inverse correlation between the hypermethylation of CpGs of *SPARC* promoter and its transcript levels was observed by analyzing 450K methylation array data for the CpG of *SPARC* for an independent cohort of 877 LUADs and 765 LUSC of TCGA dataset. Together, our data corroborate the link between methylation and expression of SPARC just observed in many other solid tumors [33,34]. 

We also found that *SPARC* methylation had a prognostic value by impacting on the overall survival of NSCLC patients. Even if this is not surprising for many methylated genes in lung cancer, the role of SPARC in this context is novel. Moreover, in solid tumors, aberrant methylation frequently involves DNA CpG dinucleotides at the 5′ end of tumor suppressor genes and it is related to the gene silencing and neoplastic process [35]. In lung cancer, this phenomenon was commonly observed during the neoplastic progression, but only more recently investigated in patients with an early stage condition of disease [36,37].

As SPARC is a protein involved in many cellular processes, such as proliferation, spreading, adhesion, motility and invasion, the silencing of *SPARC* gene in lung tumor cells could have a great impact on the neoplastic enhancement. Consistent with this hypothesis, we found that *SPARC* methylation levels in NSCLC were associated in our cohort with high methylation levels in SqCC and are able to predict patients’ survival since it identified early stage patients with significant shorter survival after surgical resection. To our knowledge, this is the first evidence of a potential utility of SPARC epigenetic silencing as prognostic marker in early stage lung cancer. 

One possible explanation of this finding comes from the previous observations that when expressed within the tumor, SPARC exerts a protective role and contrasts the aggressiveness of the tumor itself, whereas stromal SPARC supports tumor growth and tumor-stroma interaction, contributing to a more aggressive malignancy [5]. In support of this hypothesis, we observed that, under the 5-Aza-CdR treatment, the cell proliferation, invasion and migration in the lung cell line A549 were inhibited as for cell viability.

Since the identification of early markers to stratify recurrence-risk in surgically resected early stage lung patients is a significant unmet need in oncology, the *SPARC* methylation could offer an interesting clinical application in this field. *SPARC* is frequently methylated in lung cancer and not in our panel of normal samples tested; moreover, *SPARC* methylation can be easily detected in FFPE tissues by QMSP methodology and this molecular approach was able to rapidly assess the global methylation of 11 CpGs located into the main CpG island of the gene [14,31] with a sensitivity of 61% and a specificity of 100%. After further validation, such real-time PCR based test could be potentially used to predict increased risk of disease replace in patients with a possible application of this assay in a non-invasive approach, such as liquid biopsy. 

Several open questions remain to be addressed. Firstly, not all CpGs located in the promoter islands of genes could exert a significant control in the transcription in lung cancer, so the evaluation of methylation status of each single CpG of *SPARC* in non-neoplastic and tumor tissues could help to have more information about the correlation among methylation, SPARC expression and its possible role as predictive or prognostic maker in different disease stage. This investigation should also be performed by considering that many other epigenetic factors that enhance DNA methylation status could ultimately affect SPARC expression in the tumor. Secondly, the mechanisms of regulation of SPARC expression and functions in the different cell types and SPARC expression exhibits distinctive compartmentalization with differential effects on tumor and stromal cell differentiation and plasticity. For this reason, additional cell-based preclinical models with a careful interpretation of the gene expression profiling are needed. Thirdly, given that the localization of SPARC protein within NSCLC tissues is associated with disease prognosis, further studies on a larger cohort with different histologies are needed to establish all factors that regulate a heterogeneous and differential SPARC expression in NSCLC, and whether SPARC serves different functions in tumor and in the stroma. Finally, if demonstrated that *SPARC* methylation can be detected in cfDNA of NSCLC, it could represent an interesting non-invasive marker of early diagnosis of NSCLC to test in liquid biopsy or to monitoring cancer evolution in NSCLC patients. In order to prove this, an extensive validation must be performed in the light of epigenetic plasticity in normal non-neoplastic cells, the flexibility of the epigenetic factors related to external and internal factors, the heterogeneity intrinsic to the tumors.

## Figures and Tables

**Table 1:** Clinical, tumor stages and histological features of NSCLC affected patients enrolled for the study (*n* = 59).

Characteristics	TOTAL (*n* = 59)	ADC (*n* = 34)	SqCC (*n* = 25)
**Age at Diagnosis (mean yy ± SD)**	67.7 ± 8.4	67.5 ± 10.2	68.0 ± 5.2
**Gender (*n*, %)**			
Male	50 (84.8)	25 (73.5)	25 (100.0)
Female	9 (15.2)	9 (26.5)	0 (0.0)
**Smoking status (*n*, %)**			
Smoker	28 (47.5)	13 (38.2)	15 (60.0)
Never smoker	6 (10.1%)	6 (17.7)	0 (0.0)
Former smoker	25 (42.4%)	15 (44.1)	10 (40.0)
**Disease Stage**			
IA/B	28 (47.5)	18 (52.9)	10 (40.0)
IIA/B	14 (23.7)	6 (17.7)	8 (32.0)
IIIA/B	11 (18.6)	4 (11.7)	7 (28.0)
IV	6 (10.2)	6 (17.7)	0 (0.0)
**pT ^§^ (*n*, %)**			
T1	20 (33.9)	14 (41.2)	6 (24.0)
T2	27 (45.7)	14 (41.2)	13 (52.0)
T3	6 (10.2)	4 (11.7)	2 (8.0)
T4	6 (10.2)	2 (5.9)	4 (16.0)
**pN ^†^ (*n*, %)**			
N0	42 (71.2)	27 (79.4)	15 (60.0)
N1	10 (17.0)	3 (8.8)	7 (28.0)
N2	7 (11.8)	4 (11.8)	3 (12.0)
**pM (*n*, %)**			
M0	54 (91.5)	29 (85.3)	25 (100.0)
M1	5 (8.5)	5 (14.7)	0 (0.0)

ADC, adenocarcinoma; SqCC, squamous cell carcinoma; yy, years; SD, standard deviation. **^§^** pT, pathological Tumor; **^†^** pN, pathological Node.

**Table 2:** Patients’ characteristics according to *SPARC* methylation status (methylated/unmethylated).

Characteristics	SPARC Unmethylated	SPARC Methylated	*p*-Value *
***n***	23	36	
**Age at Diagnosis (mean yy ± SD)**	67.5 ± 8.7	67.8 ± 8.3	0.704
**Gender (*n*, %)**			0.726
Male	19 (82.6)	31 (86.1)	
Female	4 (17.4)	5 (13.9)	
**Smoking Status (n, %)**			0.741
Smoker	10 (43.5)	18 (50.0)	
Never smoker	3 (13.0)	3 (8.3)	
Former smoker	10 (43.5)	15 (41.7)	
**Disease Stage**			0.936
IA/B	11 (47.8)	17 (47.2)	
IIA/B	5 (21.7)	9 (25.0)	
IIIA/B	4 (17.4)	7 (19.5)	
IV	3 (13.1)	3 (8.3)	
**pT ^§^ (*n*, %)**			0.604
T1	7 (30.4)	13 (36.1)	
T2	12 (52.2)	15 (41.7)	
T3	1 (4.4)	5 (13.9)	
T4	3 (13.0)	3 (8.3)	
**pN (*n*, %)**			0.701
N0	16 (69.6)	26 (72.2)	
N1	5 (21.7)	5 (13.9)	
N2	2 (8.7)	5 (13.9)	
**pM (*n*, %)**			0.369
M0	20 (87.0)	34 (94.4)	
M1	3 (13.0)	2 (5.6)	
**Histology**			0.423
ADC	15 (65.2)	19 (52.8)	
SqCC	8 (34.8)	17 (47.2)	
**KRAS Status ^§^**			1.000
Wild-type	18 (90.0)	29 (90.6)	
Mutated	2 (10.0)	3 (9.4)	
Missing data	3	4	

ADC, adenocarcinoma; SqCC, squamous cell carcinoma; yy, years; SD, standard deviation. * *p*-values from Mann-Whitney U test or Fisher exact test for continuous and categorical variables, respectively; ^§^ patients with missing values were not considered.

**Table 3:** Distribution of *SPARC* methylation levels by NSCLC patients’ characteristics.

Characteristics	*n*	SPARC Methylation Level ^^^	*p*-Value ^#^
**Age at Diagnosis**	59	*r* = 0.10	0.454
**Gender**			0.270
Male	50	7.25 (0.00–65.60)	
Female	9	1.40 (0.00–4.30)	
**Smoking Status**			0.658
Smoker	28	4.64 (0.00–62.89)	
Former smoker	25	6.90 (0.00–64.40)	
Never smoked	6	0.70 (0.00–26.02)	
**pT ^§^**			0.279
T1	20	5.64 (0.00–35.75)	
T2	27	3.70 (0.00–65.60)	
T3	6	73.95 (21.60–173.20)	
T4	6	1.28 (0.00–7.60)	
**pN ^†^**			0.701
N0	42	4.04 (0.00–50.90)	
N1	10	6.23 (0.00–545.20)	
N2	7	7.60 (0.00–173.20)	
**pM**			0.110
M0	54	7.25 (0.00–65.60)	
M1	5	0.00 (0.00–3.53)	
**Histology**			0.053
ADC	34	3.12 (0.00–21.60)	
SqCC	25	20.60 (0.00–173.20)	
**Disease Stage**			0.282
IA/B	28	4.30 (0.00–42.68)	
IIA/B	14	47.03 (0.00–291.25)	
IIIA/B	11	4.30 (0.00–173.20)	
IV	6	1.77 (0.00–4.38)	
**KRAS Status ^§^**			0.634
Wild-type	47	4.38 (0.00–64.40)	
Mutated	5	6.90 (0.00–20.10)	

^§^ patients with missing values were not considered; ^ Spearman correlation coefficient (*r*) or median levels along with interquartile range (i.e., first-third quartiles) for continuous and categorical variables, respectively; ^#^ *p*-values from Spearman correlation or Mann-Whitney U test (or Kruskal-Wallis as appropriate) for continuous and categorical variables, respectively. **^†^** pN, pathological Node.

**Table 4:** Results from univariable Cox regression analysis that assess the association between *SPARC* methylation levels and status with overall survival and progression free survival in all NSCLC patients and within early stage tumor NSCLC patients and according to tumor histology.

Sample	Histology	Outcome	*SPARC* Methylation	SD	N. Events	N. Total	Group	HR (95%CI)	*p*-Value
All NSCLC	All	OS	Levels	194.59	31	54	-	1.46 (1.07–2.00) *	**0.018**
			Status	-	31	54	Met vs. Unmet	1.39 (0.66–2.90)	0.385
		PFS	Levels	194.59	28	51	-	0.98 (0.61–1.57) *	0.931
			Status	-	28	51	Met vs. Unmet	0.73 (0.35–1.53)	0.400
	ADC	OS	Levels	40.84	17	30	-	0.80 (0.47–1.37) *	0.420
			Status	-	17	30	Met vs. Unmet	1.38 (0.52–3.64)	0.518
		PFS	Levels	40.84	19	29	-	0.99 (0.64–1.54) *	0.968
			Status	-	19	29	Met vs. Unmet	0.84 (0.34–2.08)	0.704
	SqCC	OS	Levels	277.08	14	24	-	2.04 (1.21–3.45) *	**0.008**
			Status	-	14	24	Met vs. Unmet	1.41 (0.44–4.49)	0.566
		PFS	Levels	277.08	9	22	-	1.30 (0.61–2.75) *	0.498
			Status	-	9	22	Met vs. Unmet	0.64 (0.17–2.38)	0.503
Early tumor stage (I-II) NSCLC	All	OS	Levels	200.40	40	20	-	1.97 (1.32–2.93) *	**0.001**
			Status	-	40	20	Met vs. Unmet	1.83 (0.70–4.78)	0.217
		PFS	Levels	200.40	37	15	-	1.12 (0.51–2.44) *	0.781
			Status	-	37	15	Met vs. Unmet	0.70 (0.25–1.95)	0.499
	ADC	OS	Levels	42.45	22	11	---	1.00 (0.58–1.72) *	0.994
			Status	-	22	11	Met vs. Unmet	1.75 (0.50–6.04)	0.379
		PFS	Levels	42.45	21	11	-	0.99 (0.56–1.75) *	0.971
			Status	-	21	11	Met vs. Unmet	0.78 (0.23–2.58)	0.681
	SqCC	OS	Levels	287.42	18	9	-	2.96 (1.43–6.12) *	**0.003**
			Status	-	18	9	Met vs. Unmet	2.06 (0.43–9.93)	0.368
		PFS	Levels	287.42	16	4	-	2.03 (0.55–7.58) *	0.290
			Status	-	16	4	Met vs. Unmet	0.60 (0.08–4.29)	0.613

ADC, adenocarcinoma; SqCC, squamous cell carcinoma; OS, overall survival; PFS, progression free survival; SD, standard deviation of *SPARC* methylation level; Met, Methylated; Unmet, Unmethylated; HR, Hazard Ratio; CI, Confidence Interval. * Hazard ratio for each unitary increase of one standard deviation of *SPARC* methylation level. Bolded values refer to the significant *p*-values.

## Supplementary Materials

The following are available online at https://www.mdpi.com/2073-4409/9/6/1523/s1, Figure S1: Amplification plots for *ACTB* (**A**) and *SPARC* gene (**B**) reported in the A549 cell line by QMSP; Figure S2: Correlation analysis between *SPARC* methylation and expression values from TCGA datasets; Figure S3: Variable dependence plots show the relationship between *SPARC* methylation levels and both the overall-survival and progression-free survival; Table S1: Molecular alterations in *EGFR* and *KRAS* genes identified by Sanger sequencing in NSCLC samples.
