# Supplementary material for: Potential Prognostic Role of SPARC Methylation in Non-Small-Cell Lung Cancer
Source: Cells. 2020 Jun 22;9(6):1523. doi: 10.3390/cells9061523 (PMC7349117; doi:10.3390/cells9061523)
Supplement: Supplementary file 1 [file cells-09-01523-s001.pdf]

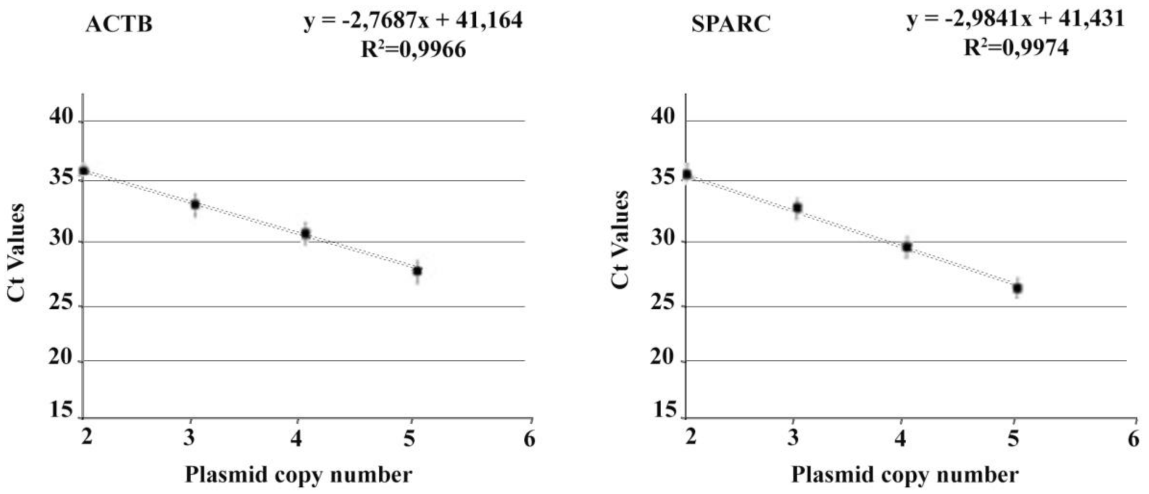

Figure S1. Amplification plots for *ACTB* (**A**) and *SPARC* gene (**B**) reported in the A549 cell line by QMSP.

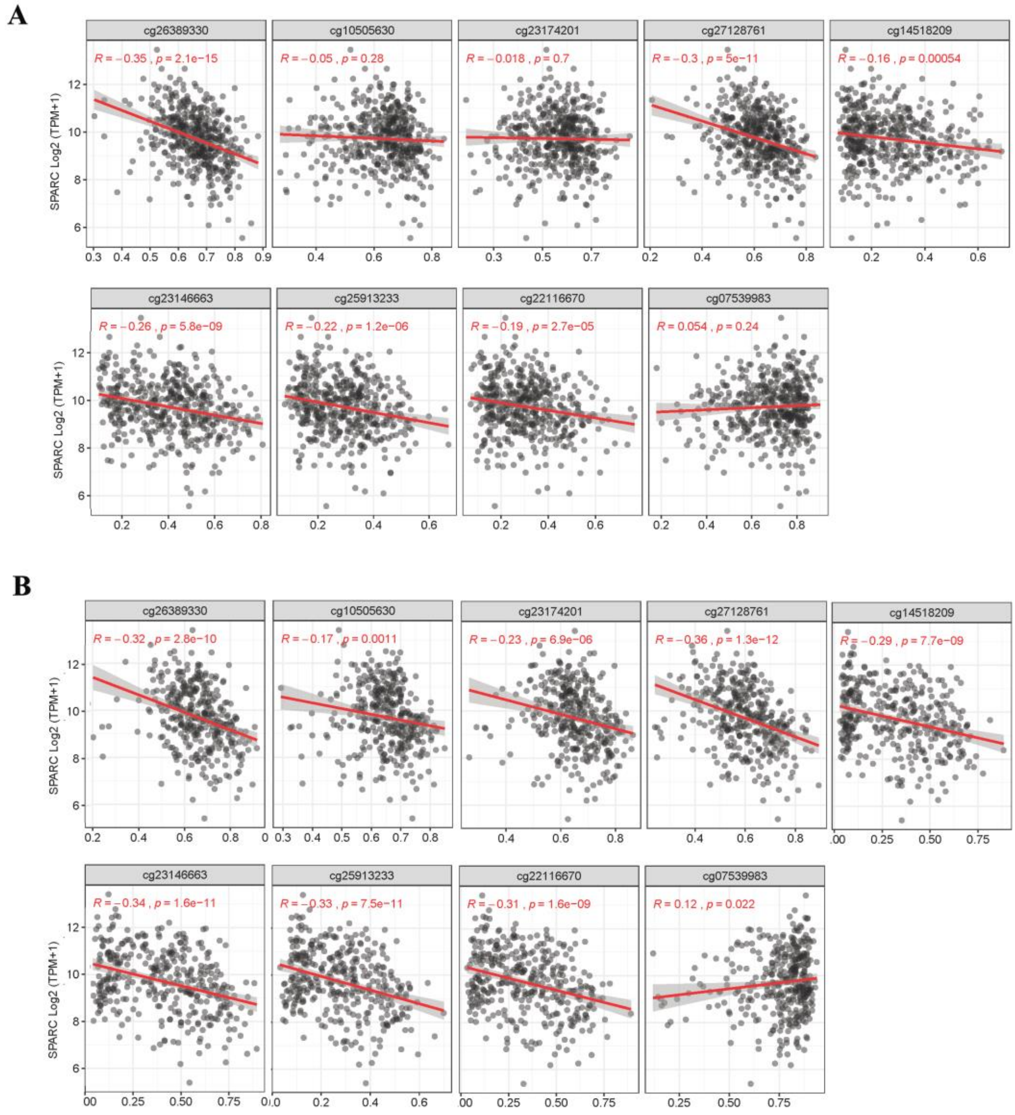

Figure S2. Correlation analysis between *SPARC* methylation and expression values from TCGA datasets.

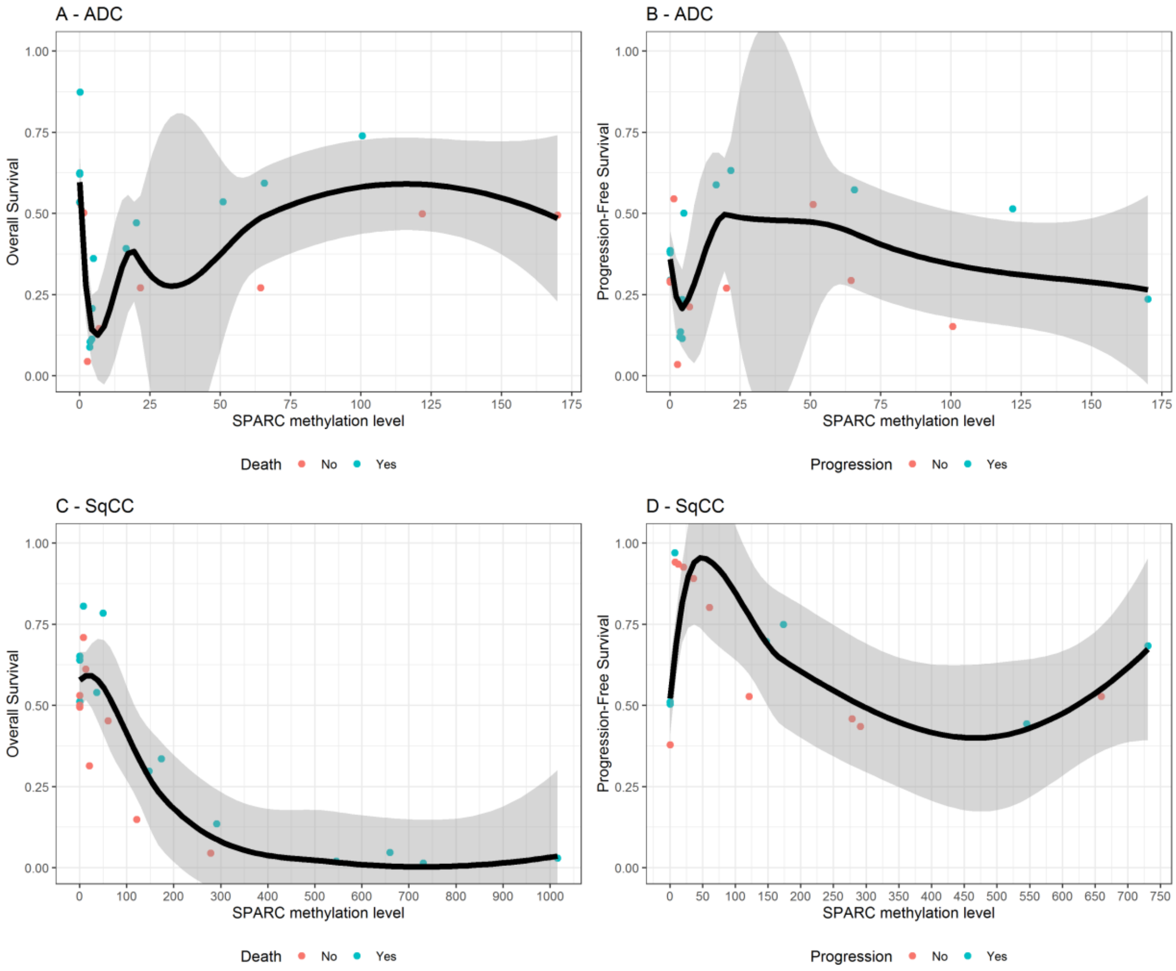

Figure 3. Variable dependence plots show the relationship between *SPARC* methylation levels and both the overall-survival and progression-free survival.

Table S1. Tumor stages, histological features and smoking habits of learning NSCLC cohort ($n = 21$).

ID cases	Gender	Smoking status	Histology	pTpNpM
LCCH-881	M	current smoker (66)	ADC	pT1cN0M0
LCCH-889	F	current smoker (40)	ADC	pT1aN2M0
LCCH-888	M	never smoker (0)	ADC	pT1bN2M0
LCCH-877	M	former smoker (32)	ADC	pT1aN0M0
LCCH-876	M	current smoker (101)	ADC	pT2aN0M0
LCCH-1027	M	current smoker (80)	SqCC	pT1bNxM0
LCCH-830	M	former smoker (40)	ADC	pT4N0M0
LCCH-773	M	former smoker (52)	ADC	pT2aN0M0
LCCH-1033	F	former smoker (40)	ADC	pT1bN2M1a
LCCH-774	F	former smoker (20)	SqCC	pT1aN0M0
LCCH-903	F	former smoker (1)	ADC	pTisN0M0
LCCH-944	M	former smoker (30)	ADC	pT1bN0M0
LCCH-1032	M	former smoker (45)	SqCC	pT4N2M0

LCCH-745	M	current smoker (80)	ADC	pT1aN0M0
LCCH-776	M	current smoker (103)	ADC	pT4N0M0
LCCH-1028	M	former smoker (32)	ADC	pT1aN1M0
LCCH-1029	M	current smoker (30)	ADC	pT3N2M0
LCCH-1030	M	current smoker (40)	ADC	pT1cNoM0
LCCH-981	M	former smoker (62)	ADC	pT4N0M0
LCCH-1031	M	former smoker (40)	ADC	pT1cN1M0
LCCH-842	M	former smoker (78)	ADC	pT1aN0M0

ADC, adenocarcinoma; SqCC, squamous cell carcinoma. () indicates pack/year number.

Table S2. Molecular alterations in *EGFR* and *KRAS* genes identified by Sanger sequencing in NSCLC samples.

ID case	Histology	*EGFR* status	*EGFR* nucleotidic change/aa change	*KRAS* status	*KRAS* nucleotidic change/aa change
LCCH-18	ADC	wt	wt	mut	c.34 G>C/p.G12R
LCCH-89	ADC	wt	wt	mut	c.34 G>T/p.G12C
LCCH-93	ADC	wt	wt	mut	c.34 G>T/p.G12C
LCCH-154	SqCC	wt	wt	mut	c.35 G>T/p.G12V
LCCH-156	ADC	wt	wt	mut	c.35 G>T/p.G12V
LCCH-473	ADC	wt	wt	mut	c.34 G>T/p.G12C

ADC, adenocarcinoma; SqCC, squamous cell carcinoma; wt, wild type; mut, mutated.
